# Key drug-targeting genes in pancreatic ductal adenocarcinoma

**DOI:** 10.18632/genesandcancer.210

**Published:** 2021-03-11

**Authors:** Meena Kishore Sakharkar, Sarinder Kaur Dhillon, Mohit Mazumder, Jian Yang

**Affiliations:** ^1^ Drug Discovery and Development Research Group, College of Pharmacy and Nutrition, University of Saskatchewan, Saskatoon, SK, Canada; ^2^ Institute of Biological Sciences, Faculty of Science, University of Malaya, Kuala Lumpur, Malaysia; ^3^ School of Life Sciences, Jawaharlal Nehru University, New Delhi, India

**Keywords:** pancreatic ductal adenocarcinoma, differentially expressed genes, protein-protein interaction network (PPI), fibronectin 1, serpin peptidase inhibitor B5

## Abstract

Pancreatic ductal adenocarcinoma (PDAC) is a highly lethal type of cancer. In this study, we undertook a pairwise comparison of gene expression pattern between tumor tissue and its matching adjacent normal tissue for 45 PDAC patients and identified 22 upregulated and 32 downregulated genes. PPI network revealed that fibronectin 1 and serpin peptidase inhibitor B5 were the most interconnected upregulated-nodes. Virtual screening identified bleomycin exhibited reasonably strong binding to both proteins. Effect of bleomycin on cell viability was examined against two PDAC cell lines, AsPC-1 and MIA PaCa-2. AsPC-1 did not respond to bleomycin, however, MIA PaCa-2 responded to bleomycin with an IC_50_ of 2.6 μM. This implicates that bleomycin could be repurposed for the treatment of PDAC, especially in combination with other chemotherapy agents. *In vivo* mouse xenograft studies and patient clinical trials are warranted to understand the functional mechanism of bleomycin towards PDAC and optimize its therapeutic efficacy. Furthermore, we will evaluate the antitumor activity of the other identified drugs in our future studies.

## INTRODUCTION

Pancreatic ductal adenocarcinoma (PDAC), which is highly lethal and makes up to more than 80% of all pancreatic cancer cases, is a type of exocrine pancreatic cancer often found in the head of the pancreas. Based on study results from the GLOBOCAN project conducted by the World Health Organization (WHO), pancreatic cancer ranks as the 12th most common cancer in the world with the age-standardized rate (ASR) for incidence and mortality at 4.2% and 4.1%, respectively [1]. Since surgical resection is still the only hope for a cure up to now, PDAC is usually treated with pancreatectomy, followed by adjuvant chemotherapy using gemcitabine or a combination of 5-flurouracil and leucovorin [2]. Pancreaticoduodenectomy (Whipple procedure) is commonly adopted to treat tumors from the head of the pancreas; whereas laparoscopic surgery is ideal to treat tumors from the tail of the pancreas [3, 4]. Although advances in surgical instruments and techniques have significantly brought down the mortality rate for the pancreaticoduodenectomy procedure, the ASR for 5-year net survival remains less than 5% for PDAC patients [5-7]. 

Poor prognosis for PDAC is normally associated with the following factors. First, pancreas is an organ positioned behind the stomach and deep in the abdomen. PDAC is almost non-palpable until being diagnosed at a late stage, which, in turn, significantly reduces the possibility of cure by pancreatectomy [8, 9]. The deep position of the pancreas also makes PDAC relatively insensitive to radiation therapy [10]. Secondly, PDAC is highly malignant, and invades and metastasizes rapidly [11, 12]. For example, Yu *et al*. showed that the progression of PDAC from stage IA to stage III with tumor size > 4 cm took only about 1 year [12]. Thirdly, PDAC patients commonly experience malnutrition and deteriorated health conditions not only because of tumor metabolism (Warburg effect) but also due to pancreatic exocrine insufficiency (*i.e.* reduced secretion of bicarbonate and digestive enzymes), making them less tolerable to chemotherapy treatment [13, 14]. Finally, PDAC is capable of generating highly dense fibrotic tissues (altered extracellular matrix constituting as high as 90% of the tumor volume) and developing hypovascularity (deficiency of blood vessels), which, in turn, would significantly hinder the delivery of chemotherapeutic agents [15, 16]. Therefore, developing more effective screening tests for early detection, identifying novel drug-design targets that are capable of decreasing the generation of fibrotic tissues and inhibit metastasis, and improving the pancreatic enzyme replacement therapy (PERT) would be highly beneficial for the survival of PDAC patients. 

Pancreatic carcinogenesis is a complex and complicated process. However, the rapid expansion of microarray and RNA-seq databases provides the possibility to systematically analyze the change of gene expression pattern during this process and identify key drug-targeting genes for pancreatic cancer. In the current study, we undertook a pairwise comparison of the gene expression pattern between PDAC tumor and its adjacent normal pancreatic tissue from 45 patients and identified *FN1* and *SERPINB5*, which encode fibronectin 1 (FN1) and serpin peptidase inhibitor B5 (Serpin B5, Maspin), respectively, as the key drug-targeting genes in developing novel therapeutic agents for PDAC. Our virtual screening showed that bleomycin and octreotide exhibit reasonably strong binding to FN1 and bleomycin, desmopressin, phosphonoacetic acid, cobicistat and oxytocin exhibit reasonably strong binding to Serpin B5, respectively. We evaluated the effect of bleomycin on cell viability of two PDAC cell lines, AsPC-1 and MIA PaCa-2, and bleomycin gave an IC_50_ of 2.6 μM towards the MIA PaCa-2 cells at 72h of treatment. However, further preclinical studies are warranted to confirm whether bleomycin, as well the other identified FDA-approved drugs, would indeed elicit its antitumor activity via FN1 and/or Serpin B5 under both in vitro and *in vivo* conditions and could be repositioned as a therapeutic agent for PDAC. 

**Figure 1:**
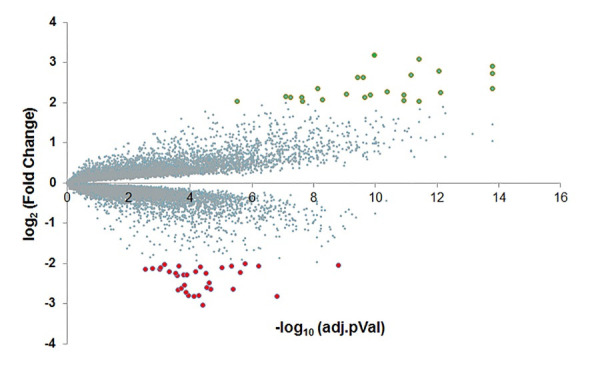
Volcano plot for the differentially expressed genes (DEGs) identified from matching pairs of tumor tissue and adjacent non-tumor tissue from 45 pancreatic ductal adenocarcinoma (PDAC) patients. The up- and down-regulated genes, significantly upregulated DEGs (log2(fold change) > 2) and significantly downregulated DEGs (log2(fold change) < -2) were shown in cadet blue, green and red dots, respectively. The top 15 up- and down-regulated genes were also listed in Table 1

**Table 1:** Table 1: Most significantly up- and down-regulated genes in human pancreatic ductal adenocarcinoma (PDAC)

Top 15 up-regulated genes				Top 15 down-regulated genes			
Gene name	Log_2_FC	P. Value	Adj. P. Val.	Gene name	Log_2_FC	P. Value	Adj. P. Val.
*CEACAM5*	3.18	2.16E-13	1.07E-10	*PNLIPRP1*	-3.03	2.37E-06	3.86E-05
*SLC6A14*	3.08	2.65E-15	3.82E-12	*PNLIPRP2*	-2.82	5.36E-06	7.56E-05
*LAMC2*	2.90	7.03E-19	1.59E-14	*IAPP*	-2.81	2.36E-09	1.53E-07
*GALNT5*	2.79	3.62E-16	8.70E-13	*CTRC*	-2.80	3.47E-06	5.30E-05
*TSPAN1*	2.73	2.49E-18	1.63E-14	*GP2*	-2.79	8.79E-06	1.12E-04
*CTSE*	2.68	6.00E-15	6.93E-12	*CEL*	-2.72	1.14E-05	1.39E-04
*POSTN*	2.63	6.76E-13	2.53E-10	*CPA2*	-2.65	2.38E-05	2.47E-04
*CEACAM6*	2.63	1.06E-12	3.78E-10	*ALB*	-2.63	1.43E-07	4.12E-06
*ANXA10*	2.36	5.23E-11	7.58E-09	*FAM24B-CUZD1*	-2.63	1.84E-05	2.01E-04
*LAMB3*	2.34	1.19E-18	1.59E-14	*ERP27*	-2.59	1.64E-06	2.88E-05
*ITGA2*	2.27	5.63E-14	4.28E-11	*CLPS*	-2.54	1.29E-05	1.53E-04
*TMPRSS4*	2.26	2.85E-16	7.48E-13	*SERPINI2*	-2.48	1.28E-06	2.38E-05
*FN1*	2.21	3.25E-12	8.61E-10	*PLA2G1B*	-2.31	2.51E-05	2.58E-04
*COL11A1*	2.19	3.05E-13	1.42E-10	*CELA2A*	-2.28	1.43E-05	1.66E-04
*SERPINB5*	2.18	1.26E-14	1.21E-11	*CELA2B*	-2.28	1.02E-05	1.26E-04

## RESULTS

### Differentially expressed genes (DEGs)

To identify novel drug-design targets for PDAC, we downloaded the microarray dataset GDS4336, which contains the gene expression data for matched tumor tissue and adjacent non-tumor tissue from 45 PDAC patients [17]. After normalization and standardization of the microarray data using the R and Affy package, we identified 54 differentially expressed genes (DEGs) using a threshold of false discovery rate (FDR) at 0.05 and log2-fold change higher than 2 (Figure 1). Out of the 54 DEGs, 22 genes are upregulated and 32 genes are downregulated. The 15 most significantly upregulated genes are *CEACAM5*, *SLC6A14*, *LAMC2*, *GALNT5*, *TSPAN1*, *CTSE*, *POSTN*, *CEACAM6*, *ANXA10*, *LAMB3*, *ITGA2*, *TMPRSS4*, *FN1*, *COL11A1* and *SERPINB5*; whereas the 15 most significantly downregulated genes are *PNLIPRP1*, *PNLIPRP2*, *IAPP*, *CTRC*, *GP2*, *CEL*, *CPA2*, *ALB*, *FAM24B-CUZD1*, *ERP27*, *CLPS*, *SERPINI2*, *PLA2G1B*, *CELA2A* and *CELA2B* (Table 1). 

### Gene ontology (GO) based functional and pathway enrichment analysis

**Figure 2:**
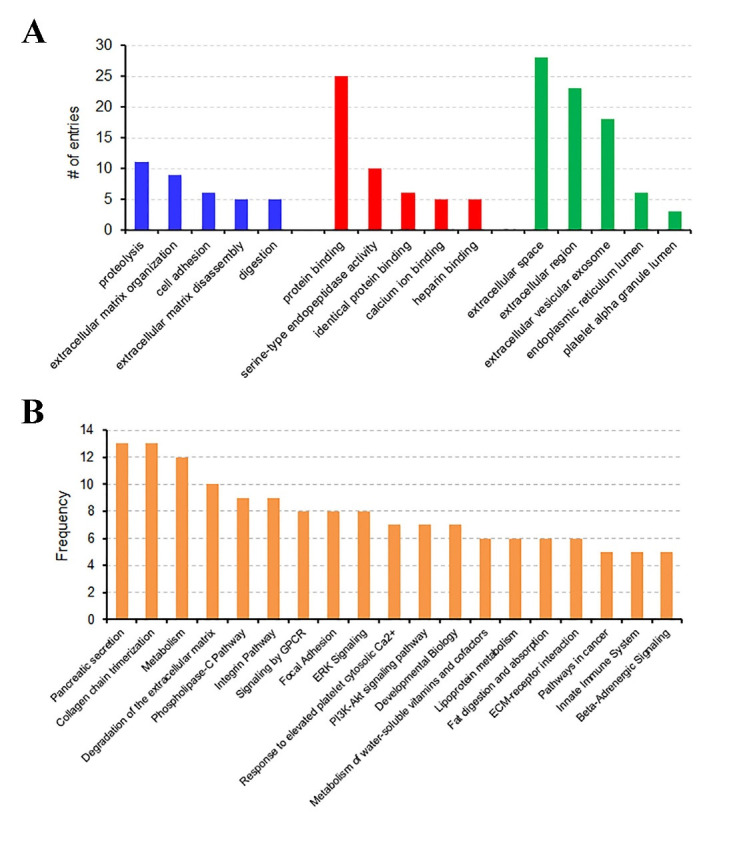
The top 5 most significantly enriched GO terms of DEGs in the categories of biological process (shown in blue), molecular function (shown in red) and cellular component (shown in green) (A) and the super pathways identified for the DEGs using GeneALaCart (B).

Gene names of the 54 DEGs were subjected to a gene ontology (GO) evaluation using FunRich [18]. GO enrichment analysis revealed large lists of enriched genes, which correspond to significant GO terms (P < 0.05), in the categories of biological process, molecular function and cellular component. As shown in Figure 2A, the top 5 GO terms in the three categories are proteolysis, extracellular matrix organization, cell adhesion, extracellular matrix disassembly and digestion; protein binding, serine-type endopeptidase activity, identical protein binding, calcium ion binding and heparin binding; and extracellular space, extracellular region, extracellular vesicular exosome, endoplasmic reticulum lumen and platelet alpha granule lumen, respectively. The DEGs associated with each biological process, molecular function or cellular component are listed in Table 2. Furthermore, we undertook the super pathway analysis of the 54 DEGs using GeneALaCart. The top 10 super pathways were identified to be pancreatic secretion, collagen chain trimerization, metabolism, degradation of the extracellular matrix, phospholipase-C pathway, integrin pathway, signaling by GPCR, focal adhesion, ERK signaling, and response to elevated platelet cytosolic Ca2^+^ (Figure 2B). 

**Table 2:** Table 2: Gene ontology (GO) functional and pathway enrichment analysis of the DEGs in human pancreatic ductal adenocarcinoma (PDAC)

***GO term***	***Genes***
***Biological process***	
Proteolysis	CTRC, CPA2, CELA2A, CELA2B, CELA3A, CTRL, CTRB2, CTRB1, CPA1, CELA3B, TMPRSS4
Extracellular matrix organization	COL1A2, ITGB6, SERPINB5, COL11A1, FN1, ITGA2, LAMB3, POSTN, LAMC2
Cell adhesion	ITGB6, FN1, ITGA2, LAMB3, POSTN, LAMC2
Extracellular matrix disassembly	CTRB2, CTRB1, FN1, LAMB3, LAMC2
Digestion	CELA3A, CTRL, CTRB2, CTRB1, CTSE
***Molecular function***	
Protein binding	IAPP, CTRC, CEL, ALB, ERP27, SERPINI2, COL1A2, PDIA2, CPA1, EGF, NR5A2, F11, CST1, KRT19, ITGB6, AGR2, SERPINB5, FN1, TMPRSS4, ITGA2, LAMB3, ANXA10, CEACAM6, POSTN, TSPAN1
Serine-type endopeptidase activity	CTRC, CELA2A, CELA2B, CELA3A, CTRL, CTRB2, CTRB1, CELA3B, F11, TMPRSS4
Identical protein binding	IAPP, ALB, COL1A2, AOX1, CLDN18, FN1
Calcium ion binding	PNLIPRP1, PNLIPRP2, PLA2G1B, EGF, ANXA10
Heparin binding	CEL, F11, FN1, POSTN, LAMC2
***Cellular component***	
Extracellular space	PNLIPRP1, PNLIPRP2, IAPP, CEL, CPA2, ALB, SERPINI2, PLA2G1B, CELA2A, CELA2B, CELA3A, CTRL, CTRB2, CTRB1, PNLIP, COL1A2, CPA1, EGF, CELA3B, F11, CST1, AGR2, SERPINB5, COL11A1, FN1, CEACAM6, POSTN, LAMC2
Extracellular region	PNLIPRP1, PNLIPRP2, IAPP, CTRC, GP2, CEL, CPA2, ALB, FAM24B, CLPS, PLA2G1B, CELA2A, CELA2B, CTRB2, CTRB1, PNLIP, COL1A2, EGF, F11, COL11A1, FN1, LAMB3, LAMC2
Extracellular vesicular exosome	GP2, CEL, ALB, CLPS, SERPINI2, COL1A2, REG1B, EGF, KIAA1324, AOX1, F11, KRT19, ITGB6, SERPINB5, FN1, CTSE, TSPAN1, SLC6A14
Endoplasmic reticulum lumen	ALB, ERP27, COL1A2, PDIA2, COL11A1, FN1
Platelet alpha granule lumen	ALB, EGF, FN1

### Protein-protein interaction (PPI) network 

**Figure 3:**
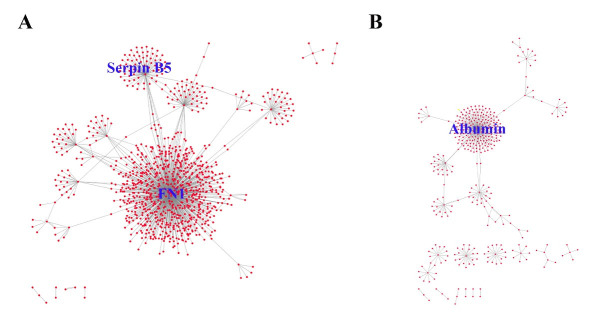
The PPI network of the significantly upregulated DEGs with FN1 (encoded by gene *FN1*) and Serpin B5 (encoded by gene *SERPINB5*) as the most connected proteins (A); and the PPI network of the significantly downregulated DEGs with albumin (encoded by gene *ALB*) as the most connected protein (B).

To get a better understanding on the biological functions of the 54 DEGs in PDAC, we extracted the partner proteins that interact with the proteins encoded by these DEGs from the BioGrid database. For the upregulated DEGs, the top 10 most connected genes are *FN1* (747), *SERPINB5* (79), *KRT19* (52), *CST1* (34), *ITGA2* (28), *LAMB3* (22), *AGR2* (21), *CEACAM6* (9), *CTSE* (8) and *ITGB6* (7), with number in brackets representing the number of interacting protein partners for each encoded DEG protein. In the case of downregulated DEGs, the top 10 most connected genes are *ALB* (187), *COL1A2* (23), *NR5A2* (23), *PDIA2* (21), *EGF* (18), *CELA2B* (15), *CELA3A* (12), *PNLIP* (10), *PNLIPRP1* (9) and *CPA2* (8). As shown in Figure 3, FN1 (encoded by gene FN1) and Serpin B5 (encoded by gene *SERPIN5*) are the top two most-connected proteins for the upregulated DEGs and albumin (encoded by gene *ALB*) is the most-connected protein for downregulated DEGs. 

### Structural modeling and drug virtual screening 

**Figure 4:**
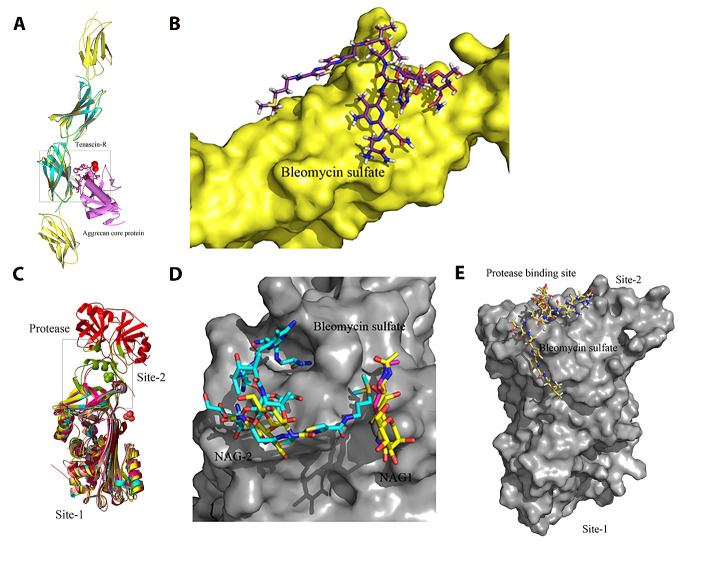
Structural modeling of FN1 (4-domain fragment) and Serpin B5 and docking of bleomycin sulfate, an approved anticancer drug, to FN1 and Serpin B5. **(A)**. A cartoon representation of the superimposition of the 4-domain fragment of FN1, containing the RGD loop and synergy site, and the complex structure of tenascins and aggrecan lectin domain. **(B)**. Potential binding mode of bleomycin sulfate at the mapped interaction site between FN1 and aggrecan core protein after docking simulations. **(C)**. A carton representation of the superimposition of the crystal structure of Serpin B5 and the crystal structures of its homologues to identify the protease binding sire (Site 2). **(D)**. Potential binding mode of bleomycin sulfate at the N-acetyl-D-glucosamine (NAG) binding site (Site 1) of Serpin B5 after docking simulations. **(E)**. Potential binding mode of bleomycin sulfate at the protease binding site (Site 2) of Serpin B5 after docking simulations.

Using the multi-template homology modeling technique, we constructed a fragment of FN1 consisting of four domains and screened virtually for potential drugs in four databases (Selleck, DiscoveryProbe FDA-approved Drug Library, DrugBank and Binding Database) at the binding interface between FN1 and aggrecan core protein (Figure 4A). Bleomycin and octreotide exhibit reasonably strong binding to FN1. For Serpin B5, we screened virtually for drugs from the four databases at the N-acetyl-D-glucosamine (NAG) binding site (Site 1) and the protease binding site (Site 2) (Figure 4C). Bleomycin, desmopressin, phosphonoacetic acid, cobicistat and oxytocin exhibit reasonably strong binding to Serpin B5. 

### Effect of bleomycin on cell viability of AsPC-1 and MIA PaCa-2 cells 

**Figure 5:**
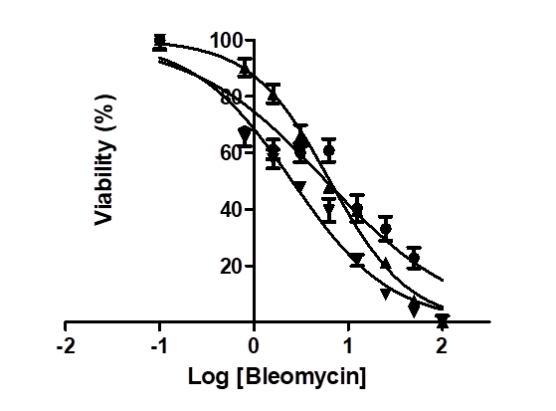
The effect of bleomycin on cell viability of MIA PaCa-2 cells at treatment time of 24 h (●), 48 h (▲) and 72 h (▼), respectively. The IC_50_ of bleomycin was measured to be 5.9 μM for 24 h of treatment, 6.4 μM for 48 h of treatment, and 2.6 μM at 72 h of treatment, respectively.

Effect of bleomycin on cell viability of two PDAC cell lines, AsPC-1 and MIA PaCa-2, was measured at treatment time of 24 h, 48 h and 72 h, respectively. AsPC-1 cells did not respond to bleomycin sulfate; however, cell growth of MIA PaCa-2 was significantly suppressed by bleomycin with an IC_50_ of 5.9 μM at 24 h of treatment, 6.4 μM at 48 h of treatment, and 2.6 μM at 72 h of treatment, respectively (Figure 5). 

## DISCUSSION

As one of the deadliest types of cancer, PDAC is commonly diagnosed in late stages and is associated with rapid progression and metastasis, resulting in the ASR for 5-year net survival less than 5% [5, 6, 19, 20]. Due to the lack of effective therapy, cure is seldom considered as a treatment outcome while efforts are mainly intended to prolong the survival of PDAC patients. Most of the research studies on PDAC are aimed at identifying methods for accurate early diagnosis, increasing net survival, improving quality of life, and implanting more effective palliative care for PDAC patients, in addition to continued efforts directed at searching for novel therapeutic agents. The rapid expansion of microarray and RNA-seq data from PDAC patients has provided us a treasure of information to be utilized for better understanding on the molecular basis for carcinogenesis, progression and metastasis of PDAC and develop novel therapeutic and/or supportive treatment options to improve the clinical outcome for PDAC patients. 

In a previous study, *Shi et al*. screened for DEGs and constructed a co-expression network to identify dysregulated pathways for PDAC using microarray dataset GSE15471, which consists of the gene expression data of matched tumor tissue and adjacent non-tumor tissue from 36 PDAC patients [21]. They identified 766 up-regulated DEGs and 170 down-regulated DEGs using a lower cut-off standard of |log_2_(fold change)| >1. The dysregulated pathways, which might significantly contribute to the development and progression of PDAC, were identified to be mainly involved in immune response, homeostasis and cell adhesion. However, the list of DEGs was not released and no detailed analysis on any individual DEG was performed. Using the same microarray dataset, Sartor *et al*. screened for master regulators (MRs) of transcription and highlighted the potential value of having tubby-like protein 3 (TULP3) as a clinical prognostic biomarker for PDAC [22]. In the current study, we used a different microarray dataset, GDS4336, which contains matched tumor tissue and adjacent non-tumor tissue from 45 PDAC patients. As illustrated in Figure 2 and Table 2, most of the DEGs are present outside the cells and involved in the extracellular matrix (ECM) organization, disassembly and regulation. ECM is an important structural component of human cells and exerts its biological functions in gene expression, signal transduction, cell adhesion, cell migration, cell invasion and angiogenesis [23-27]. Remodeling of ECM has been shown to enable the infiltration of tumor cells into the pancreas and surrounding tissues such as lymphatic organs and peritoneum, which subsequently leads to cancer metastasis to the liver and lungs [28, 29]. Since altered ECM can constitute as high as 90% of the tumor’s volume, developing novel therapeutic agents or repurposing drugs targeting the above-identified DEGs to suppress both cancer cell growth and tumor ECM formation would be beneficial for PDAC patients. 

**Figure 6:**
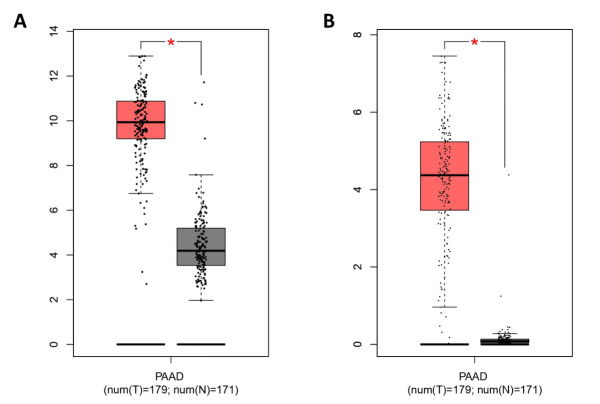
Boxplots showing the expression of gene *FN1* (A) and *SERPINB5* (B) in PDAC patients versus normal controls in patient dataset PAAD deposited in The Cancer Genome Atlas (TCGA). This figure was generated using online software GEPIA (http://gepia.cancer-pku.cn).

**Figure 7:**
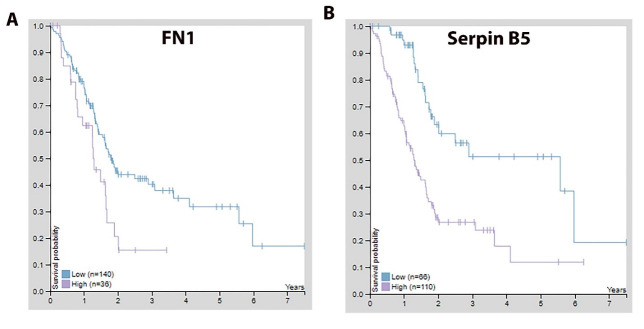
Kaplan-Meier plots of the patient survival probability versus the mRNA expression level of *FN1* (A) and *SERPINB5* (B) based on the patients’ data deposited at the Human Protein Atlas (HPA) database (http://www.proteinatlas.org). Both *FN1* (p = 0.014) and *SERPINB5* (p < 0.001) were identified to be unfavorable prognostic factors for PDAC.

When we examined the ECM-related proteins (Table 2), FN1 and Serpin B5 emerged as the top two nodes with a high degree of connectivity in the PPI network for upregulated DEGs (Figure 3A). Hence, they are highly likely to be biological hubs that regulate important physiological and/or pathophysiological functions in PDAC development and progression. Our current results were consistent with previous studies [30, 31]. Since biological hubs, such as MDM2 oncoprotein, are usually selected as potential drug design targets [32, 33], we decided to investigate further on FN1 and Serpin B5. FN1, which is a multi-domain protein encoded by gene *FN1* located at the breast cancer susceptible locus 2q35, is actively engaged in cell adhesion and ECM organization and disassembly. It facilitates the crosstalk between tumor environment and cancer cells [34]. FN1 has been shown to be upregulated in different types of cancer and promotes cancer growth, progression, invasion and metastasis [35-38]; and thus, is proposed as a target for cancer imaging and treatment [39]. Serpin B5, which is a 42 kDa serine protease inhibitor encoded by gene *SERPINB5* located at band 18q21.33, is proposed as a tumor suppressor and found to be downregulated in breast cancer [40-42]. Recently, the expression of Serpin B5 was shown to be positively correlated with overall and progression-free survival in gastric cancer patients [43]. However, the expression of Serpin B5 is reported to be upregulated in pancreatic adenocarcinoma, endometrial cancer and ovarian carcinoma, suggesting that Serpin B5 plays an oncogenic rather than tumor-suppressing role [44-47]. As shown in Table 2, Serpin B5 is involved in ECM organization; and this might contribute to oncogenic function of Serpin B5 in PDAC. We further reconfirmed the overexpression of genes *FN1* and *SERPINB5* in pancreatic adenocarcinoma patients using dataset PAAD deposited in The Cancer Genome Atlas (Figure 6). Subsequently, we searched the Human Protein Atlas (HPA) database to explore whether FN1 or Serpin B5 could be used a prognostic indicator for PDAC. The predicted survival probability is much higher for pancreatic cancer patients with low mRNA expression of *FN1* or *SERPINB5* (Figure 7). This implicated that FN1 and Serpin B5 are valid drug-design targets for PDAC. 

Surprisingly, our drug virtual screening against four databases (Selleck, DiscoveryProbe FDA-approved Drug Library, DrugBank and Binding Database) identified that bleomycin sulfate (Blexane, Blenoxane), a mixture of cytotoxic glycopeptide antibiotics approved for squamous cell carcinoma, lymphoma, testicular carcinoma and malignant pleural effusion, exhibited reasonably strong binding to both FN1 and Serpin B5 (Figure 4 and Supplementary Table S1 and Figure S1). This implies that bleomycin sulfate could be repurposed for the treatment of PDAC. Cell viability assay showed that MIA PaCa-2, instead of AsPC-1, responded to bleomycin treatment with an IC_50_ of 2.6 μM at 72 h of treatment (Figure 5). This is consistent with previous studies by Girelli *et al*. [48], showing that bleomycin exhibited an IC_50_ of 3.5 μM towards MIA PaCa-2 cells and electroporation could further bring the IC_50_ down to 0.2 μM. In addition, Ichor Medical Systems Incorporated (San Diego, CA, USA) initiated a phase I clinical trial on *Electroporation therapy with bleomycin in treating patients with pancreatic cancer* in December 2000, but later withdrew the clinical trial in September 2012 without any actual enrollment (https://clinicaltrials.gov/ct2/show/study/NCT00027521). At the present time, it is unclear why AsPC-1 was not responding to bleomycin treatment, and we are also unable to confirm whether bleomycin indeed inhibited the growth of MIA PaCa-2 cells *via* FN1 and/or Serpin B5. Further studies are warranted on whether bleomycin would suppress the growth of other PDAC cell lines (both *in vitro* and *in vivo*), whether it indeed binds to FN1 and/or Serpin B5, how it regulates the biological functions of these two proteins in both PDAC cells and tumor ECM, and whether it can inhibit DNA synthesis in PDAC. 

Finally, the results of PPI network for the downregulated DEGs clearly differentiated albumin (encoded by gene *ALB*) as a key component of the network (Figure 3B). Albumin is an important plasma protein carrier for hormones, fatty acids, metabolites and drugs. Along with hypovascularity, decreased expression of albumin would worsen pancreatic exocrine insufficiency and chemotherapeutic drug delivery in PDAC patients. The U.S. Food and Drug Administration (FDA) has approved an albumin-stabilized nanoparticle formulation of paclitaxel (Abraxane) for pancreatic cancer, and a very recent study by Zhao *et al*. showed that an albumin-binding-5-fluorouracil prodrug exhibited longer half-life (t_1/2_) and enhanced antitumor activity in a rodent model of hepatoma [49]. Thus, we propose a novel intravenous regimen consisting of albumin and chemotherapeutic drugs such as 5-fluorouracil and gemcitabine, interspersed with pancreatic enzyme replacement therapy (PERT), to treat PDAC. 

## MATERIALS AND METHODS

### Microarray data

The microarray dataset GDS4336 was downloaded from Gene Expression Omnibus (GEO), which was based on the platform of Affymetrix Human Gene 1.0 ST Array. The dataset GDS4336 contains matching pairs of tumor tissue and adjacent non-tumor tissue from 45 pancreatic ductal adenocarcinoma (PDAC) patients [17].

### Differentially expressed genes (DEGs) 

The original CEL data were imported into the R (version 3.4.4) and Affy packages for background correction and normalization. After normalization and standardization, differentially expressed genes (DEGs) were identified between matching pairs of tumor tissue and adjacent non-tumor tissue from 45 PDAC patients using Limma from R. The P value of 0.05 and |log2(fold change)| > 2 was chosen as the cut-off standard (Figure 1). 

### Gene ontology and pathway enrichment analysis 

The identified DEGs were subjected to gene ontology (GO) evaluation using FunRich under P < 0.05. GO enrichment analysis revealed large lists of enriched genes in the categories of biological process, molecular function and cellular component. The top five GO terms in each of the three categories were summarized in Figure 2A. The DEGs were then sent to GeneALaCart (https://genealacart.genecards.org) for super pathway analysis, and the identified super pathways were shown in Figure 2B.

### Protein-protein interactions (PPI) 

PPI networks were constructed for both upregulated- and downregulated-DEGs using BioGRID (Version 3.4.155) and then visualized using Cytoscape (The Cytoscape Consortium, https://www.cytoscape.org). In the PPI networks (Figure 3), the proteins are regarded as “notes”, and the number of interactions of a protein is defined as the degree of that note. The notes with high degree of connectivity are defined as “hubs”, which are usually considered as potential drug design targets. 

### Virtual screening for potential drugs targeting FN1 and Serpin B5 

Structural model of FN1 bound with the aggrecan core protein was generated using protein homology/comparative modeling using a fragment of FN1 encompassing Type-III repeats 7-10 (PDB code: 1FNF) [50]. The virtual screening was undertaken at the interface between FN1 and aggrecan core protein. For Serpin B5, two potential binding sites, the N-acetyl-D-Glucosamine binding site (site1) and the protease binding site (site 2), were identified upon superimposing the structure of Serpin B5 (PDB code: 1WZ9) [51] with various homologous proteins. The virtual screening was undertaken at both sites of Serpin B5. The ligand library was prepared by downloading molecules from four free chemical databases (Selleck, DiscoveryProbe FDA-approved Drug Library, DrugBank and Binding Database). Molecular docking of the ligand library was performed within a grid box of 20 Å in each dimension at the three binding sites using software Schrödinger (Schrödinger, LLC, New York, NY, USA).

### Cell viability assay 

Effect of bleomycin on cell viability of PDAC cell lines AsPC-1 and MIA PaCa-2 was determined with the MTT assay using our published protocol [52]. Briefly, either AsPC-1 or MIA PaCa-2 cells were plated in 96-well plates at 10,000 cells per well with final cell culture volume of 100 μL. The cells were allowed to grow to 70-80% confluence before being treated with bleomycin (concentration range: 0.1 – 100 μM) for 24 h, 48 and 72 h, respectively. 

## CONCLUSION

In the current study, we identified 54 DEGs, 22 upregulated genes and 32 downregulated genes, from a pairwise gene expression comparison for 45 PDAC patients. FN1 and Serpin B5 were identified as the most interconnected nodes in the PPI network for upregulated DEGs and likely to be important targets for novel drug development and drug repurposing. Our drug virtual screening identified that bleomycin exhibited reasonably strong binding to both FN1 and Serpin B5, and the subsequent cell viability assay showed that PDAC cell line MIA PaCa-2 responded to bleomycin with an IC_50_ of 2.6 μM. Further studies are warranted to confirm whether the inhibitory activity of bleomycin is indeed *via* FN1 and/or Serpin B5.

## SUPPLEMENTARY MATERIALS TABLES AND FIGURES


