# Supplementary material for: Key drug-targeting genes in pancreatic ductal adenocarcinoma
Source: Genes Cancer. 2021 Mar 11;12:12–24. doi: 10.18632/genesandcancer.210 (PMC8045979; doi:10.18632/genesandcancer.210)

SUPPLEMENTARY MATERIALS

Table S1. Docking of bleomycin sulfate to fibronectin 1 (FN1) and Serpin B5

Protein	Docking score	XP score	Glide energy	Hydrogen bond	Hydrophobic interactions
FN1 (fragment)	-7.521	-8.506	-106.4	Ile1333,Asp1334, Glu1364, Gln1412, Gln1413, Ser1414	Phe1335, Ile1338, Glu1392, Val1394, Leu1408, Ile1410, Gly1411, Thr1415
Serpin B5 (Site 1)	-8.111	-9.851	-141.7	Lys47 Asp49, Thr50, Thr301, Asp303, Ser305, Ser308, Thr310, Gly312	Gly48, Ser101, Glu103, His295, Phe304, Glu309, Lys311, Val313
Serpin B5 (Site 2)	-6.263	-7.248	-93.4	Lys181, Glu201, Lys270, Ser272	Glu177, Ser178, Glu179, Thr180, Asn199, Lys268, Lys275, Glu328, Glu347

Figure S1. The Ligplots showing the potential binding interactions of bleomycin sulfate at the interaction site between FN1 and aggrecan core protein (**A**), the NAG-binding site of Serpin B5 (**B**), and the protease binding site of Serpin B5 (**C**), respectively.

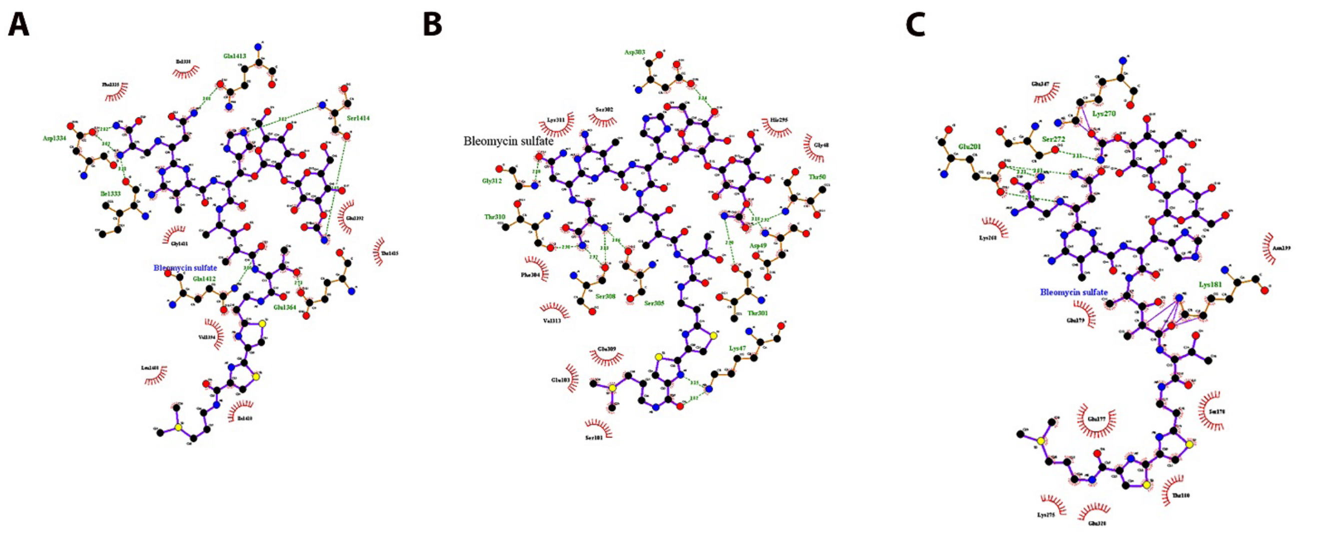

Supplement: Supplementary file 1 [file ganc-12-12-s001.pdf]
